# Unmasking the Dark Triad: A Data Fusion Machine Learning Approach to Characterize the Neural Bases of Narcissistic, Machiavellian and Psychopathic Traits

**DOI:** 10.1111/ejn.16674

**Published:** 2025-01-22

**Authors:** Richard Bakiaj, Clara Isabel Pantoja Muñoz, Andrea Bizzego, Alessandro Grecucci

**Affiliations:** ^1^ Department of Psychology and Cognitive Sciences (DiPSCo) University of Trento Trento Italy; ^2^ Center for Medical Sciences, CISMed University of Trento Trento Italy

**Keywords:** Dark Triad, personality traits, structural brain networks, transposed independent vector analysis, unsupervised machine learning

## Abstract

The Dark Triad (DT), encompassing narcissism, Machiavellianism and psychopathy traits, poses significant societal challenges. Understanding the neural underpinnings of these traits is crucial for developing effective interventions and preventive strategies. Our study aimed to unveil the neural substrates of the DT by examining brain scans from 201 individuals (mean age: 32.43, 105 females) using the unsupervised learning algorithm transposed independent vector analysis (tIVA). tIVA, known for identifying complex patterns in neuroimaging data, detected 15 joint grey matter (GM) and white matter (WM) networks. Of these networks, four were associated with the DT. The first component comprises areas within the reward network, including the thalamus, caudate, anterior cingulate and prefrontal regions. The second component encompasses regions within the executive network, predominantly involving prefrontal and posterior areas. The third component includes regions within the default mode network (DMN), such as the angular gyrus, the precuneus and the posterior cingulate cortex. Lastly, the fourth component overlaps with areas of the visual network, primarily located in the occipital and temporal lobes. Within these networks, the reward‐related component correlated with narcissism, suggesting an association with the need for constant interpersonal rewards to enhance self‐esteem and grandiosity in narcissistic individuals. Conversely, the DM‐related component correlated with Machiavellianism, potentially reflecting the heightened strategic thinking employed by Machiavellian individuals for manipulation purposes. In line with established trends, sex differences emerged, with males displaying notably higher DT scores. Our findings offer insights into the intricate neurobiological bases of the DT personality and hold implications for future research and interventions.

## Graphical abstract

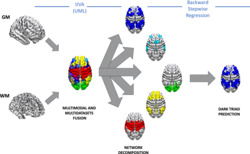

**Abbreviations**
ACC: anterior cingulate cortex
BSS: blind source separation
CAT12: Computational Anatomy Toolbox
DD: Dirty Dozen
dlPFC: dorsolateral prefrontal cortex
DMN: default mode network
DT: Dark Triad
DTI: diffusion tensor imaging
DWI: diffusion‐weighted imaging
fMRI: functional magnetic resonance imaging
GM: grey matter
ICA: independent component analysis
MACH‐IV: Machiavellianism IV Scale
MNI: Montreal Neurological Institute
MP2RAGE: Magnetization Prepared 2 Rapid Acquisition Gradient Echoes
mPFC: medial prefrontal cortex
MRI: magnetic resonance imaging
NIBS: non‐invasive brain stimulation
NPI: Narcissistic Personality Inventory
OFC: orbitofrontal cortex
PCC: posterior cingulate cortex
PFC: prefrontal cortex
SD: standard deviation
SD3: Short Dark Triad
sMRI: structural magnetic resonance imaging
SPM12: statistical parametric mapping
SRP‐III: Self‐Report Psychopathy Scale
tIVA: transposed independent vector analysis
WM: white matter

## 1 Introduction

The term Dark Triad coined by Paulhus and Williams (2002) refers to three distinct personality characteristics linked to norm‐breaking, transgressive behaviour and manipulative tendencies (McDonald, Donnellan, and Navarrete 2012): narcissism, Machiavellianism and psychopathy. The extreme manifestations of these three underlying structures of the Dark Triad take a high toll in our society both when they are considered separately and together, accounting for burdensome financial, socioecological and psychological costs. For example, the engagement of psychopathic individuals in criminal activity causes societal monetary losses for almost $500 billion (Kiehl and Hoffman 2011). Furthermore, psychopathy has been found to be associated with a preference for volatile sexual encounters (Jonason et al. 2011; Jonason, Li, and Czarna 2013), which undermine the establishment of a romantic connection and may lead to unhappy relationships (Jonason, Li, and Buss 2010). Narcissism is associated with personal gains in the short term and with social detriments in the long term (Campbell et al. 2005). Finally, a study on a large Chinese sample (Zhu, Wang, and Geng 2021) showed how some of the components of Machiavellianism, such as distrust, were responsible for the unhealthy lifestyle conducted by individuals high on this trait.

Although they share some common characteristics, each trait of the Dark Triad has its own origins and definitions. Narcissism is characterized by a grandiose sense of entitlement, supremacy and superiority on the one hand and by a vulnerable sense of sensitivity, insecurity and inadequacy on the other hand, threatening interpersonal relationships (Cain, Pincus, and Ansell 2008; Jornkokgoud et al. 2023; Pincus, Cain, and Wright 2014). Machiavellians can be ruthless, cynic, deceptive and manipulative, exploiting others for short‐term personal advantage and lacking socio‐emotional comprehension and empathy (Ali, Sousa Amorin, and Chamorro‐Premuzic 2009; Barlow, Qualter, and Stylianou 2010; Brewer and Abell 2017; Rauthmann and Kolar 2013). Psychopathy is a severe personality disorder characterized by a constellation of detrimental traits (Glenn and Raine 2014). Traditionally, two overarching factors have been associated with psychopathy (Hare 2003): Factor 1, referring to the Interpersonal‐Affective facets and including callous, manipulative and lack‐of‐guilt tendencies, and Factor 2, referring to the Lifestyle‐Antisocial facets and including lack of self‐control and risk‐taking and antisocial behaviour (Glenn and Raine 2014; Hare 1996; Hare et al. 2000; Kiehl 2006). Recent perspectives on psychopathy view it as a spectrum of traits, ranging from subclinical to clinical levels. The Triarchic Model of Psychopathy (Patrick, Fowles, and Krueger 2009) identifies three core dimensions: boldness, meanness and disinhibition. Boldness involves traits like confidence, emotional stability and fearlessness. Meanness includes emotional detachment, callousness and a tendency to exploit others. Disinhibition is marked by impulsivity, recklessness and a propensity for externalizing behaviours. These dimensions are present to varying extents in existing psychopathy models and measures, such as the traditional two‐factor model (Patrick 2022). As clinical science increasingly adopts a dimensional approach to understanding and assessing personality disorders (APA 2013), a thorough understanding of subclinical traits can enhance the literature on personality pathology and improve the conceptualization of psychopathic traits.

These three Dark Triad traits have been measured separately, using the Machiavellianism‐IV Scale (MACH‐IV; Christie and Geis 1970), the Narcissistic Personality Inventory (NPI; Raskin and Hall 1979; Raskin and Terry 1988) and the Self‐Report Psychopathy Scale (SRP‐III; Paulhus, Neumann, and Hare 2009) to assess Machiavellianism, narcissism and psychopathy, respectively. Combining these three scales, two measures have been established to assess the three traits of the Dark Triad: the Dirty Dozen scale (DD; Jonason and Webster 2010), which includes just 12 items, and the Short Dark Triad (SD3; Jones and Paulhus 2014), which includes 27 items. These brief measures have the advantage of investigating each trait simultaneously while also reducing the amount of time needed for completion. Despite the fact that both measures have been demonstrated to have some significance in terms of statistical correlations with the individual scales (i.e., NPI, MACH‐IV and SRP‐III), the SD3 is more preferable and reliable in this respect (Maples, Lamkin, and Miller 2014).

Considering the neurobiological correlates of the Dark Triad, no existing study, to the best of our knowledge, has tried to investigate them as a single component. Only a few studies have looked at some of its sub‐components separately, leading to a still inchoate discovery of their underlying anatomical and functional alterations in the brain. For what concerns Machiavellianism, Verbeke et al. (2011) found that individuals high on this trait displayed significant variations in the basal ganglia, prefrontal cortex (PFC), insula, right hippocampus and left parahippocampal gyrus as opposed to individuals low on this trait. The basal ganglia, including the caudate and putamen, are crucial for learning, memory, social cognition and motivated behaviour (Davidson and Irwin 1999; Lieberman 2000; Verbeke et al. 2011). The PFC, particularly the orbital frontal cortex (OFC), is vital for planned social behaviour, decision‐making under uncertainty and emotional regulation (Elliott, Dolan, and Frith 2000; Lieberman 2007; Ohira et al. 2006; Tremblay and Schultz 1999). The insula has been demonstrated to processes emotional suppression, social risk decisions and risk perception (Ochsner et al. 2002; Preuschoff, Quartz, and Bossaerts 2008). The hippocampus is essential for memory formation and retrieval, with distinct involvement also noted in psychopathic behaviour (Laakso et al. 2001). All these are characteristics linked to Machiavellian traits (Verbeke et al. 2011). By the same token, in an MRI study exploring social cognition, Nestor et al. (2013) found that higher scores on the MACH‐IV were linked to more grey matter (GM) volume in the left lateral orbital gyrus.

Regarding narcissism, functional and structural alterations of insular regions seem to play an important role in the symptomatology exhibited by narcissistic individuals. As a matter of fact, the anterior insula has been established as a critical component of the neural network associated with empathy (Decety and Lamm 2006; Singer and Lamm 2009), which has been proposed to be diminished and dysfunctional in complex ways (Baskin‐Sommers, Krusemark, and Ronningstam 2014; Hart, Hepper, and Sedikides 2018; Urbonaviciute and Hepper 2020), if not lacking at all (Kernberg 1970; Kohut 1966), in narcissistic individuals. In this respect, Fan et al. (2011) found that reduced deactivation in the right anterior insula was correlated with increased levels of narcissism in an empathy condition during an fMRI study. Another study (Schulze et al. 2013), investigating 17 narcissistic personality disordered patients by using voxel‐based morphometry, found decreased GM volume in frontal‐paralimbic brain regions compared to healthy controls. In a recent investigation conducted by Jornkokgoud et al. (2023), the researchers employed machine learning techniques to delve into the structural configuration of the brain in individuals exhibiting narcissistic personality traits. The study revealed that a neural circuit encompassing the lateral and middle frontal gyri, the angular gyrus, Rolandic operculum and Heschl's gyrus effectively predicted the presence of narcissistic personality characteristics. The frontal gyrus and all the related areas have been shown to be linked to empathy and cognitive emotional processes (Li et al. 2020; Schulze et al. 2013). The Rolandic operculum has been associated with different disorders such as borderline personality disorder and addiction‐related disorders and plays a role in emotion processing and bodily self‐consciousness, potentially influencing emotional instability in narcissistic traits (Blefari et al. 2017; Schulze et al. 2013; Zhang et al. 2021). The angular gyrus is crucial for higher level social cognition and empathy and is implicated in abnormal self‐image and self‐other distinctions in narcissism (Decety and Lamm 2007; Ruby and Decety 2004). Lastly, the Heschl's gyrus, including the primary auditory cortex, is associated with auditory perception, language processing and possible abnormal internal dialogues in borderline personalities, with structural abnormalities linked to cognitive impairments and addiction‐related disorders (Karaali et al. 2016; Kasai et al. 2003; Zhang et al. 2021).

In psychopathic individuals, brain alterations have been observed as volume reductions and GM thinning in frontal, temporal and subcortical regions (Yang, Raine, Colletti, et al. 2009; Yang, Raine, Narr, et al. 2009; Yang et al. 2005). Specifically, the dorsolateral prefrontal cortex (dlPFC) and the orbitofrontal cortex (OFC) show significant structural and functional reductions compared to controls (De Oliveira‐Souza et al. 2008; Müller et al. 2008; Rilling et al. 2007). These prefrontal areas are crucial for decision‐making (Elliott, Dolan, and Frith 2000; Glenn and Raine 2014), understanding others' emotional states (Shamay‐Tsoory et al. 2005), regulating emotions (Ochsner et al. 2002), processing reward and punishment (Rolls 2000) and inhibiting responses (Völlm et al. 2006). Additionally, Muller et al. (2008) found GM reductions in the right superior temporal gyrus and the angular gyrus in psychopaths. The superior temporal gyrus is important for social cognition, perspective‐taking, mental state attribution and emotional attention (Müller et al. 2008), whereas the angular gyrus is involved in experiencing guilt and embarrassment, emotions that deter future antisocial behaviour (Glenn and Raine 2014; Takahashi et al. 2004). Subcortical regions, including the amygdala, parahippocampal gyrus, hippocampus and posterior cingulate, also show alterations in psychopathic individuals, affecting emotion regulation and empathy‐related functions (Ermer et al. 2013; Yang, Raine, Colletti, et al. 2009). Psychopathy is associated with deficits in responding to aversive stimuli, aversive conditioning, startle reflex augmentation, recognizing fearful expressions and experiencing moral emotions, all functions thought to rely on the functioning of the amygdala (Glenn and Raine 2014). Choy, Raine, and Schug (2022) recently found increased striatal volume in adult psychopaths, linked to traits such as reward seeking and impulsivity (Glenn and Raine 2014). Moreover, psychopathic individuals exhibit abnormal activation in the default mode network (DMN; Freeman et al. 2015; Juarez, Kiehl, and Calhoun 2012; Pujol et al. 2012), which is typically active during rest and self‐referential processing (Buckner and Carroll 2007; Northoff et al. 2006) but deactivates during externally‐focused tasks to engage ‘task‐positive’ networks with minimal interference (Raichle et al. 2001).

However, despite the efforts to map Machiavellianism, psychopathy and narcissism onto the brain, mixed and puzzling results hinder our understanding of how the brain is organized in individuals displaying all these traits. For example, Cope et al. (2012) found that the anterior cingulate cortex (ACC) was affected in people with psychopathic characteristics, whereas Glenn et al. (2010) had not found this result in a previous study. Similarly, the extent to which the activity of regions associated with empathy and emotion regulation (e.g., insula) is altered in narcissists is still unclear given their complex relationship (Baskin‐Sommers, Krusemark, and Ronningstam 2014). By the same token, whereas Bereczkei et al. (2013; 2015) found a heightened activation in the ACC of individuals with high Machiavellian traits during a trust game and interpreted it as the sign of an augmented emotional conflict between social norms and personal interest, Verbeke et al. (2011) did not find any structural, GM abnormality in the ACC in their study.

The mixed results in previous research may stem from methodological limitations. First, previous studies have predominantly relied on the use of mass‐univariate statistical techniques, which take into account alterations within single voxels rather than among multiple voxels. However, the contemporary understanding of brain functioning considers psychiatric and neurological symptomatology as associated with distributed patterns of voxel alterations rather than with isolated voxel anomalies (Biswal et al. 2010; Fox et al. 2005; Kennedy and Courchesne 2008; Sheffield and Barch 2016). Additionally, previous studies have relied on group‐level statistics, not allowing for drawing inferences at the individual level (Grecucci et al. 2022; Lapomarda, Grecucci, et al. 2021; Lapomarda, Pappaianni, et al. 2021; Scarpazza et al. 2020). Finally, the contribution of the white matter (WM) to the Dark Triad has yet to be considered.

Affective neuroscientists have recently shifted their attention towards the adoption of machine learning techniques. These techniques have the advantage to explore the intricate relationships among multiple brain voxels compared to traditional statistical methods, detecting subtle and distributed alterations in brain structure and function (Grecucci et al. 2022; Hebart and Baker 2018; Vieira, Pinaya, and Mechelli 2020). In other words, machine learning goes beyond assessing each individual voxel and comprehensively examines the relationship of multiple voxels simultaneously (Grecucci et al. 2022; Hebart and Baker 2018).

One type of machine learning methods is unsupervised learning, which allows researchers to unveil concealed patterns in complex datasets for a deeper understanding of large‐scale brain networks (Vieira, Pinaya, and Mechelli 2020). Unsupervised learning can also exploit data fusion strategies (Baggio et al. 2023), which, combining mutual information from different modalities such as GM and WM concentration, offer a holistic view of brain structure and function. Multivariate machine learning approaches hold promise for advancing our understanding of the complexities of the brain and its relationship with affective and personality processes.

From a methodological standpoint, the main purpose of this paper was to provide new evidence on the neural bases of DT traits by using unsupervised machine learning in the form of dimensionality reduction applied to GM and WM features in a data fusion perspective in combination with backward stepwise regression statistics. The idea was to use unsupervised learning to decompose the whole brain into independent circuits of covarying GM and WM and then to predict which brain circuit explained the DT. This way, reducing the number of features (e.g., voxels) had the advantage to limit demanding computations, ignore redundancy and only focus on meaningful information and reduce the risk of overfitting (Guyon and Elisseeff 2003).

First, unsupervised machine learning in the form of transposed independent vector analysis (tIVA) (Adali, Levin‐Schwartz, and Calhoun 2015a, 2015b; Lee et al. 2008) was used to decompose the brain into independent neural circuits. This brain decomposition method allows for a reduction of the dimensionality of the state space (≃10^5^ brain voxels) into a few naturally grouping brain networks (Ghomroudi, Scaltritti, and Grecucci 2023; Grecucci et al. 2022). These networks are more biologically plausible than atlas‐based brain parcellations based on anatomical and histological features not based on structural or temporal covariation (regions that work together form a specialized network with a common spatial and temporal profile). This approach is in accordance with the fact that personality traits are not ascribed to one specific region in the brain, but they are distributed across different networks of brain areas (Biswal et al. 2010; Fox et al. 2005; Kennedy and Courchesne 2008; Sheffield and Barch 2016). In addition, the decision to include both GM and WM components in our analyses is motivated by the fact that they both can be informative and influenced by similar genetic factors (Baggio et al. 2023; Spalletta, Piras, and Gili 2018). As a matter of fact, pathological processes affect WM as well and are not only limited to GM (Spalletta, Piras, and Gili 2018). WM studies often use diffusion tensor imaging (DTI), which is a technique that, measuring the microstructural displacement of water molecules, assesses the integrity of WM fibres (Assaf and Pasternak 2007; Alba‐Ferrara and de Erausquin 2013). However, DTI is extremely sensitive to noise, and this might result in poor reproducible regions of interest (Radwan et al. 2022). In this regard, the relevance of adopting a combined GM–WM approach resides in the fact that considering pure WM concentrations has the advantage to evaluate distributed WM alterations without the restrictions imposed by specific tracts (Baggio et al. 2023).

We predicted that three brain circuits specifically could explain Dark Triad scores on the SD3: a circuit with areas at least partly overlapping with the reward network, primarily including the ACC, the OFC, the basal ganglia, the amygdala, the hippocampus and the thalamus (Haber and Knutson 2010); a circuit with areas at least partly overlapping with the executive network, primarily including the dlPFC and the lateral posterior parietal cortex; and a circuit with areas at least partly overlapping with the DMN, primarily including the medial PFC (mPFC), the posterior cingulate cortex (pCC), the precuneus and the angular gyrus (Raichle et al. 2001). We hypothesized these brain circuits based on their potential relevance to the DT traits and their associated functions in reward processing, executive control and thinking processes, which are aspects that we find deficient in individuals displaying these personality traits.

Furthermore, we did an exploratory analysis to examine whether the three networks predicted could be linked to specific subscales of the SD3. We expected to find a correspondence between narcissistic traits and specific brain regions coinciding with those within the reward network. Likewise, we expected to observe a correlation between Machiavellian traits and brain areas overlapping with the DMN. Additionally, we expected to identify a relationship between psychopathic traits and brain regions that intersect with both the reward and executive networks. This delineation of the expected associations aims to enhance the characterization of how distinct DT traits may be represented at a neurological level. As a matter of fact, we aim to advance our understanding of the neural correlates of the DT, both as a single and separate component, by considering the unique contribution of both GM and WM.

Additionally, we investigated how age and gender would affect these three networks in the brain. Previous studies (Cale and Lilienfeld 2002; Grijalva et al. 2015; Krampen et al. 1990; Nicholls et al. 2005) have consistently found that all the sub‐traits associated with the DT are more prevalent in males as opposed to females. Although these studies have examined sex differences in each specific DT sub‐trait, there remains a gap in understanding whether there are overall sex disparities in the DT as a collective construct. We expected to find a discernible sex‐based disparity in the collective manifestation of the DT traits, indicating an elevated presence of these traits in males relative to females.

## 2 Materials and Methods

### 2.1 Participants

Participants recruited for this study were 214 German native speakers, selected from the ‘MPI‐Leipzig Mind‐Brain‐Body’ database (OpenNeuro Dataset, https://openneuro.org, accession number ds000221; Babayan et al. 2020; Harvard Dataverse 2020), which consists of structural and functional MRI and behavioural data from 321 German‐speaking people. Participants of the ‘MPI‐Leipzig Mind‐Brain‐Body’ database were selected according to specific selection criteria (see below), including completion of the SD3, medical eligibility for magnetic resonance sessions and absence of past or present psychiatric and neurological disorders. Seventeen participants were excluded for the present study because they were 70 or older years of age to avoid possible ageing alteration effects, leading to a final sample of 201 participants (105 females) between the ages of 20 and 69 (*M* = 32.43, *SD* = 13.92).

#### 2.1.1 Exclusion Criteria

- History of psychiatric diseases that required inpatient treatment for longer than 2 weeks within the last 10 years (e.g., psychosis, attempted suicide and post‐traumatic stress disorder);
- History of neurological disorders (incl. multiple sclerosis, stroke, epilepsy, brain tumour, meningoencephalitis, and severe concussion);
- History of malignant diseases;
- Intake of one of the following medications:
  ○ Any centrally active drugs (including *Hypericum perforatum* )
  ○ Beta‐ and alpha‐blocker
  ○ Cortisol
  ○ Any chemotherapeutic or psychopharmacological medication;
- Positive drug anamnesis (extensive alcohol, MDMA, amphetamines, cocaine, opiates, benzodiazepine and cannabis);
- Extensive testing experience at the MPI‐CBS or other academic institution;
- Past or present student of psychology;
- MRI exclusion criteria
  ○ Any metallic implants, braces and non‐removable piercings
  ○ Tattoos
  ○ Pregnancy
  ○ Claustrophobia
  ○ Tinnitus
  ○ Surgical operation in the last 3 months

*Note:* Adapted from Mendes et al. (2019).

Written informed consents were obtained by all participants, who received financial compensations for their participation. The study protocol was approved by the ethics committee of the University of Leipzig (097/15‐ff) (154/13‐ff) (Babayan et al. 2019; Mendes et al. 2019).

### 2.2 SD3 Questionnaire

The SD3 is a shorter and combined version of the MACH‐IV, the NPI and the SPR‐III and was created to assess the DT by focusing on the definitions and aspects of the three distinct traits (Jones and Paulhus 2014; Paulhus and Jones 2015). It is composed of 27 items—nine items for each of the three traits that form the DT—that can be rated on a 5‐point Likert scale (1 being *strongly disagree* to 5 being *strongly agree*) (Jones and Paulhus 2014). Examples of the items are ‘Make sure your plans benefit you, not others’ for Machiavellianism, ‘I know I am special because everyone tells me so’ for narcissism and ‘People who mess with me always regret it’ for psychopathy. For our purpose, the English version of the SD3 was translated into German. Reliability analyses for the SD3 were satisfactory, having obtained *Cronbach's alpha* of 0.68 for Machiavellianism (English original: α = 0.78), *Cronbach's alpha* of 0.65 for narcissism (English original: α = 0.77) and *Cronbach's alpha* of 0.59 for psychopathy (English original: α = 0.80). SD3 total scores were calculated by summing the scores of the individual DT traits.

The descriptive statistics for each dimension of the SD3 are presented in Table S1. The means, standard deviations, standard errors, minimums, maximums, ranges and 95% confidence intervals for each dimension are reported.

Paulhus (https://www.psytoolkit.org/survey‐library/short‐dark‐triad.html) proposed the normal ranges for each of the three traits (Table S2). The descriptive statistics revealed mean scores of 20.74 for Machiavellianism, 24.50 for narcissism and 18.66 for psychopathy. When comparing these scores to the normal ranges in Table S2, adjusted to account for the summing of 9 items per dimension, we see that our sample's scores fall within the normal range. Specifically, the scaled normal ranges for Machiavellianism, narcissism and psychopathy are mean scores of 27.9, 25.2 and 21.6 respectively, with corresponding thresholds for being outside the normal range at scores of 34.74 for Machiavellianism, 33.12 for narcissism and 30.6 for psychopathy. Given these comparisons, our participants' mean suggests that they exhibit normal levels of these traits.

### 2.3 MRI Data Acquisition

T1‐weighted images were acquired using a 3‐T Siemens MAGNETOM Verio scanner (Siemens Healthcare GmbH, Erlangen, Germany), which was built with a 32‐channel head coil. The data collection took place at the Day Clinic for Cognitive Neurology of the University Clinic Leipzig and the Max Planck Institute for Human and Cognitive and Brain Sciences (MPI CBS) in Leipzig, Germany. The original MPI‐Leipzig Mind‐Brain‐Body dataset comprised structural magnetic resonance imaging (sMRI), functional magnetic resonance imaging (fMRI) and diffusion‐weighted imaging (DWI) scans (Babayan et al. 2019), but, for the purposes of our study, we just considered the T1‐weighted images. The high‐resolution structural image was acquired by using a 3D MP2RAGE sequence (Marques et al. 2010) consisting of the following parameters: sagittal acquisition orientation, one 3D volume with 176 slices, TR = 5000 ms, TE = 2.92 ms, TI1 = 700 ms, TI2 = 2500 ms, FA1 = 4°, FA2 = 5°, pre‐scan normalization, echo spacing = 6.9 ms, bandwidth = 240 Hz/pixel, FOV = 256 mm, voxel size = 1 mm isotropic, GRAPPA acceleration factor 3, slice order = interleaved, duration = 8 min 22 s.

### 2.4 Preprocessing

After conducting a quality check on the images in order to exclude artefacts and before running any analyses, all data were preprocessed with the same pipeline using the segmentation operations offered by the Computational Anatomy Toolbox (CAT12, http://www.neuro.uni‐jena.de/cat/), a toolbox available for the Statistical Parametric Mapping software (SPM12, https://www.fil.ion.ucl.ac.uk/spm/software) in the MATLAB environment. This way, after reorienting each image and placing the anterior commissure as the origin, segmentation of GM, WM and cerebrospinal fluid was computed. The registration was computed utilizing Diffeomorphic Anatomical Registration through Exponential Lie algebra tools (DARTEL) (Ashburner 2007), which is a whole‐brain‐approach alternative to SPM12. Finally, DARTEL images were normalized to the MNI space using a spatial Gaussian smoothing of 12 (full width at half maximum of Gaussian smoothing kernel [12, 12, 12]) (Pappaianni et al. 2017).

### 2.5 tIVA

tIVA is a blind source separation (BSS) method, which provides a fully multivariate approach and enables fusion of data from multiple modalities, such as GM and WM and then decompose the brain into joint GM‐WM profiles (Adali, Levin‐Schwartz, and Calhoun 2015a, 2015b). The tIVA algorithm allows for the extraction of brain circuits that are maximally dependent across modalities while being independent among themselves within a modality, and this makes tIVA a more suitable algorithm when the correlation among components across modalities are significantly different as opposed to other solutions (e.g., joint independent component analysis; Adali, Levin‐Schwartz, and Calhoun 2015a, 2015b). We applied tIVA to structural data by using the Fusion ICA Toolbox (FIT, http://mialab.mrn.org/software/fit) (Calhoun et al. 2006) in the MATLAB 2018a environment (https://it.mathworks.com/products/matlab.html) (*MATLAB (R2018a)*) in order to compute the network decomposition, which consisted of separating the brain into naturally grouping circuits. Then, an estimation of the number of components for both modalities was conducted following default parameters. To investigate the reliability of each modality, the ICASSO (Himberg and Hyvärinen 2003; Himberg, Hyvärinen, and Esposito 2004) and the Infomax algorithm were used. The resulting output consisted of a matrix with the number of subjects (rows) and the loading coefficients for each component (columns). Loading coefficients represent how each component is expressed for every subject; for example, considering GM concentration, loading coefficients refer to how much GM concentration each subject has in each component compared to an average across the brain. Subsequently, the groupICA software (https://trendscenter.org/software/) was used to convert the independent components into Talairach coordinates in order to specify the brain areas associated with them. Finally, we took into consideration brain areas with both higher (positive values) and lower (negative values) GM and WM concentration and plotted them in Surf Ice (https://www.nitrc.org/projects/surfice/) (Rorden) (using a different template for GM and WM).

### 2.6 Stepwise Regression Analysis

**FIGURE 1:**
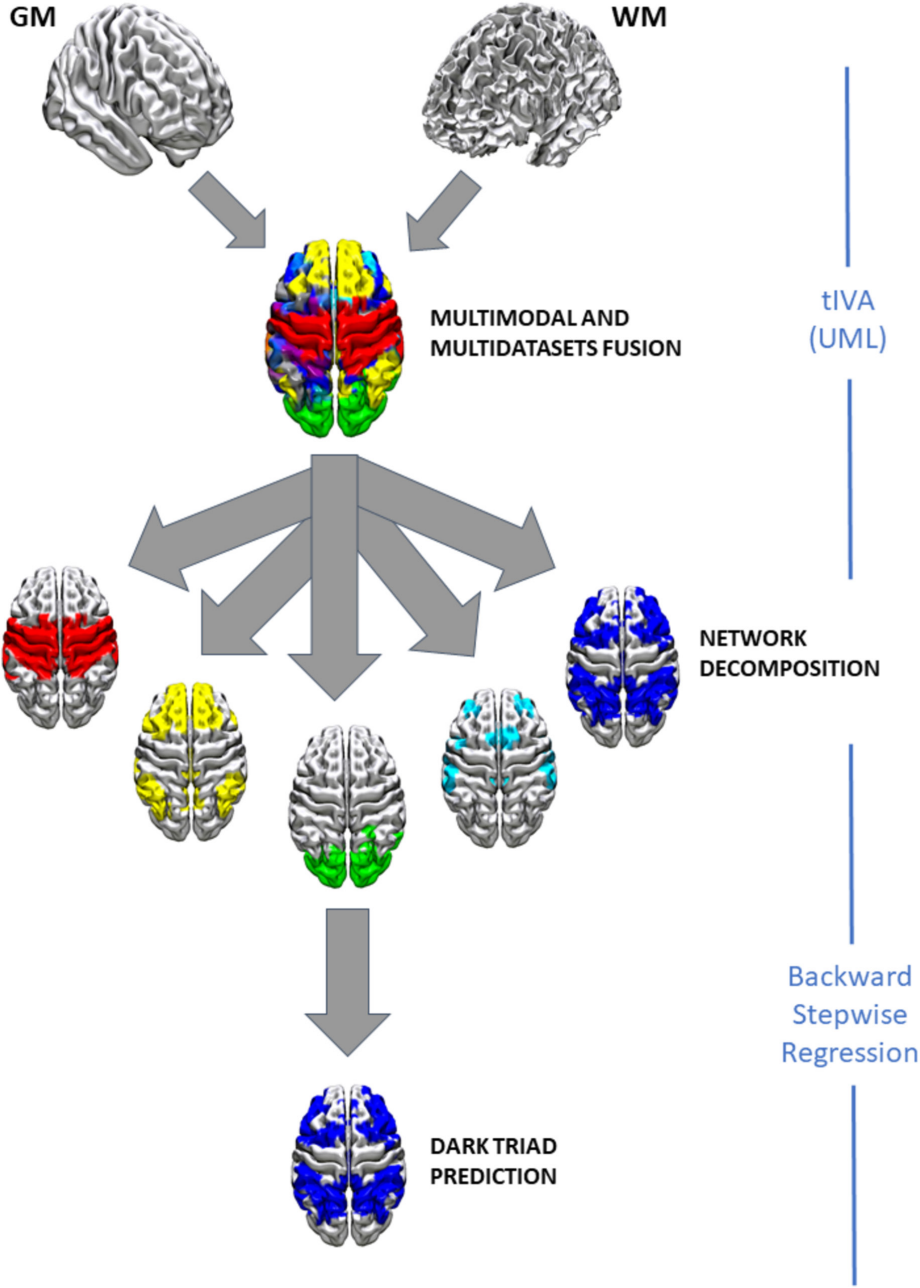
Unsupervised machine learning tIVA was used to combine the two modalities (GM and WM) and to decompose the brain into the covarying GM‐WM independent circuits. Then backward stepwise regression was applied to predict DT scores.

After having decomposed the brains of our participants in spatially independent circuits, we entered the loading coefficients of each network for each participant in a regression model to predict DT scores from the identified brain networks. A backward stepwise regression model was used in JASP (*Jasp Team (Version 0.16.2)*, 2022). All predictors were initially entered simultaneously and, then, were removed sequentially based on predefined criteria. The criteria for predictor entry were determined by a significance level of *p* < 0.05, whereas the removal criterion was set at a threshold of 0.1. Figure 1 shows a simplified representation of the pipeline (tIVA + regression) used to decompose the brains of our participants and predict the personality traits.

## 3 Results

### 3.1 Unsupervised Machine Learning to Decompose the Brain Into Covarying GM–WM Circuits

The tIVA algorithm detected 15 distinct GM and WM brain networks that were independent yet correlated. These networks were derived in a data‐driven manner, allowing for the decomposition of participants' brains into individual components using the information theoretic criteria (Wax and Kailath 1985). Each estimated GM component had a corresponding estimated WM component that exhibited a similar pattern of concentration. Positive values in these networks indicated increased concentration of GM/WM, whereas negative values suggested decreased concentration. Fundamentally, the covariation between GM and WM components implied that when GM concentration was high in a specific circuit (e.g., tIVA‐GM12), the corresponding WM concentration in that circuit (tIVA‐WM12) was also high.

### 3.2 Backward Stepwise Regression

**FIGURE 2:**
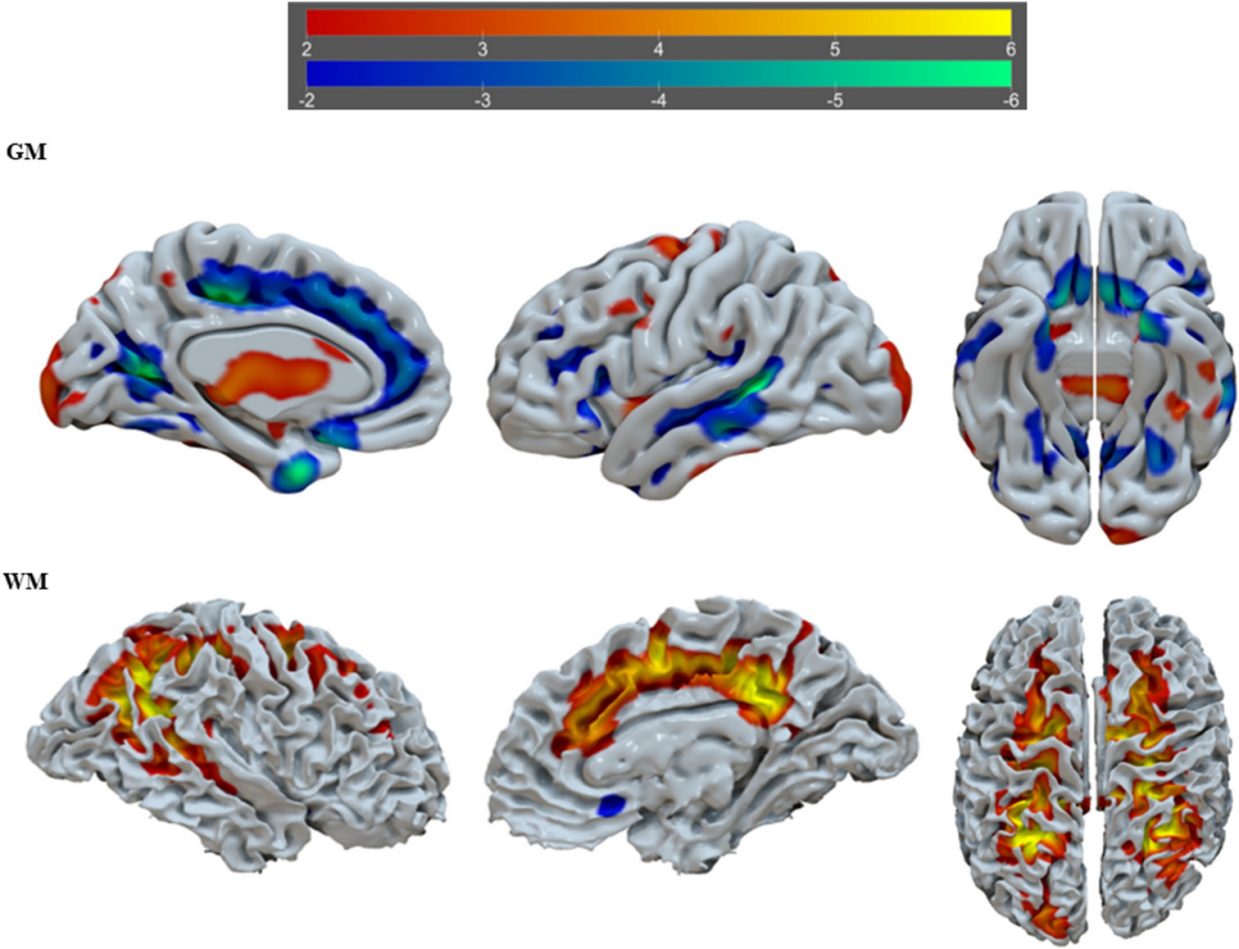
tIVA‐4 threshold was set at z‐score > 2. From left to right are displayed brain plots of tIVA‐GM 4 of the left hemisphere in medial view, of the left hemisphere in lateral view and of both hemispheres in inferior view (top) and brain plots of tIVA‐WM 4 of the right hemisphere in lateral view, of the right hemisphere in medial view and of both hemispheres in superior view. Regions with increased GM and WM are represented with warm colours, whereas regions with decreased GM and WM are represented with cold colours.

**FIGURE 3:**
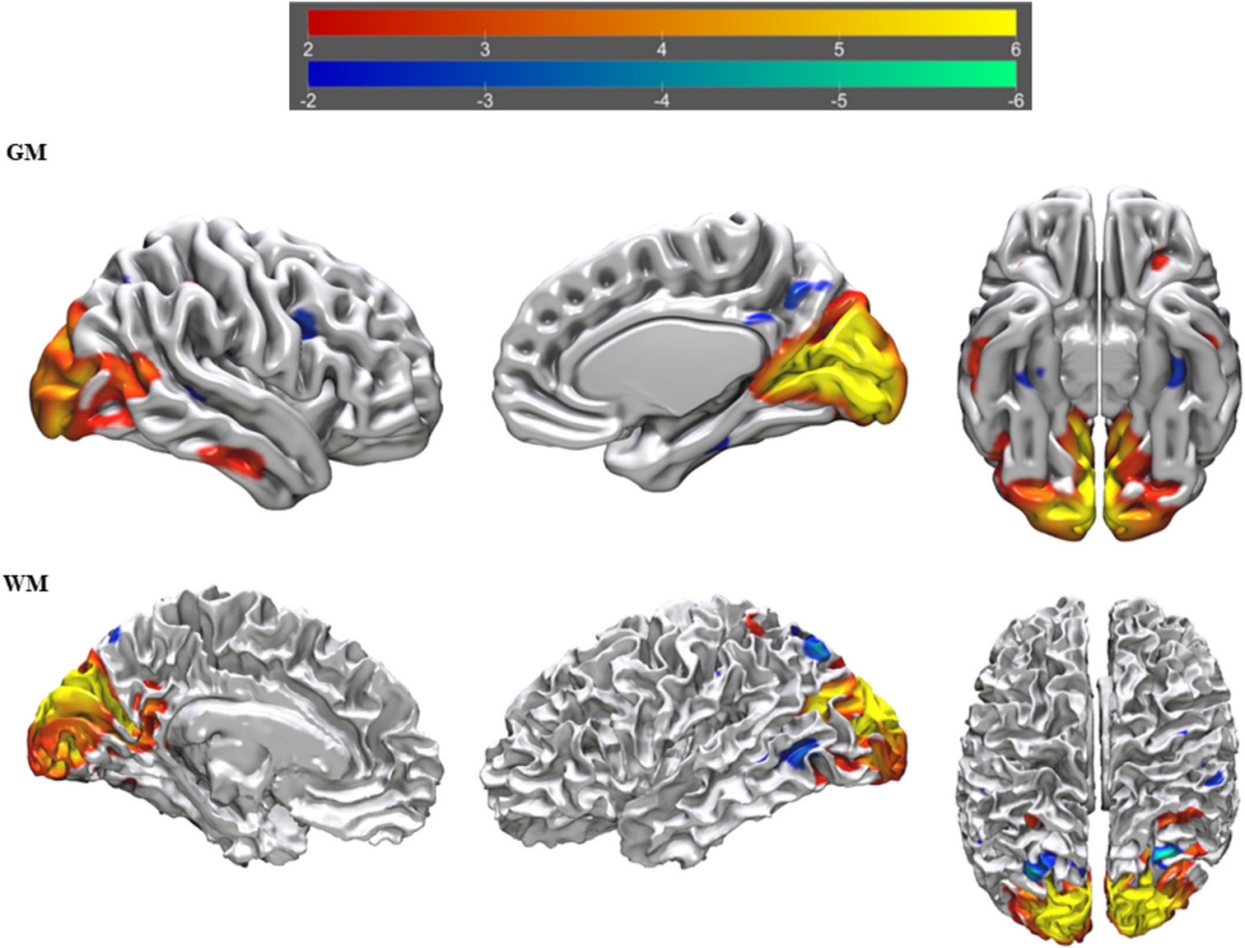
tIVA‐5 threshold was set at z‐score > 2. From left to right are displayed brain plots of tIVA‐GM 5 of the right hemisphere in lateral view, of the right hemisphere in medial view and of both hemispheres in inferior view (top) and brain plots of tIVA‐WM 5 of the left hemisphere in medial view, of the left hemisphere in lateral view and of both hemispheres in superior view. Regions with increased GM and WM are represented with warm colours, whereas regions with decreased GM and WM are represented with cold colours.

**FIGURE 4:**
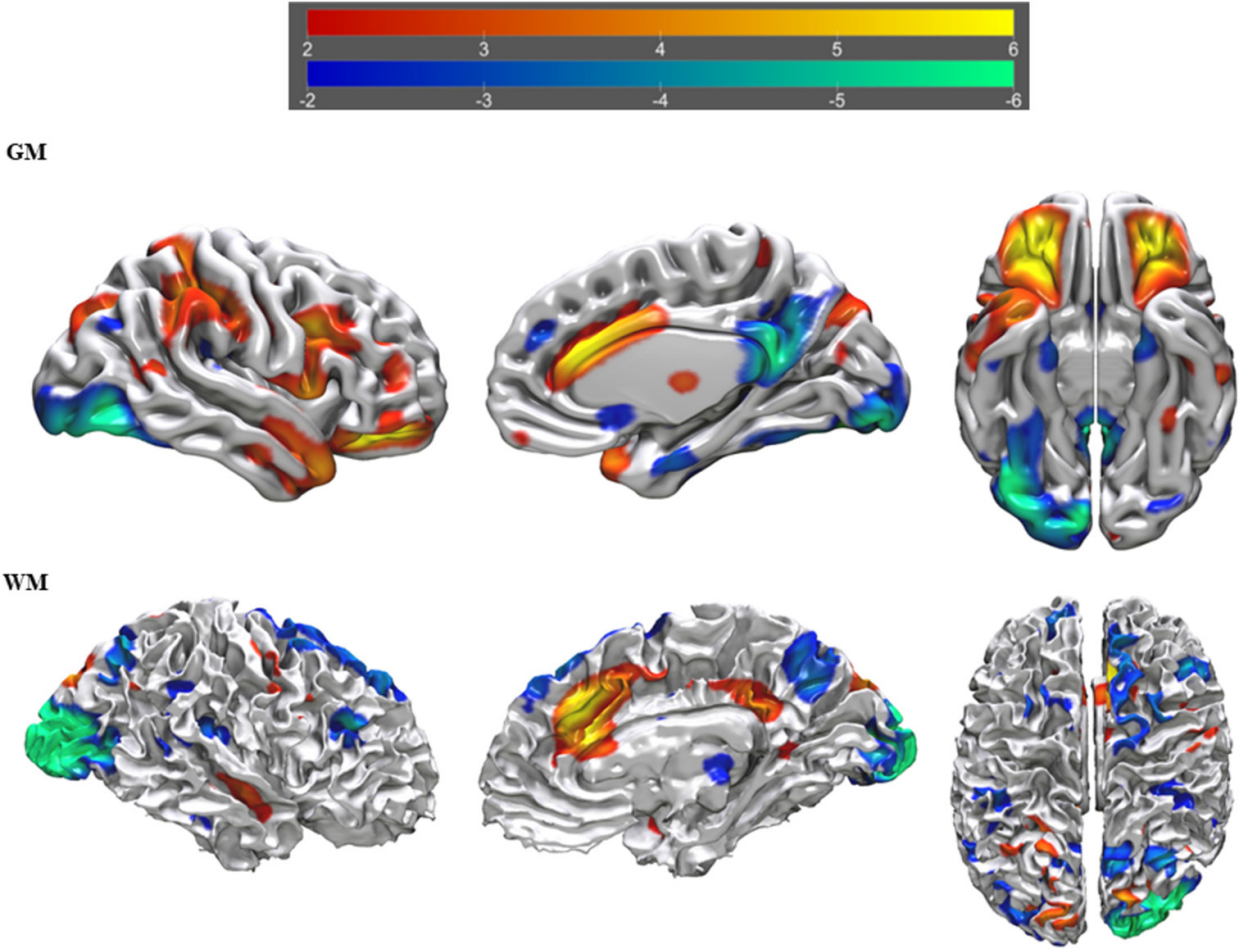
tIVA‐12 threshold was set at z‐score > 2. From left to right are displayed brain plots of tIVA‐GM 12 of the right hemisphere in lateral view, of the right hemisphere in medial view and of both hemispheres in inferior view (top) and brain plots of tIVA‐WM 12 of the right hemisphere in lateral view, of the right hemisphere in medial view and of both hemispheres in superior view. Regions with increased GM and WM are represented with warm colours, whereas regions with decreased GM and WM are represented with cold colours.

**FIGURE 5:**
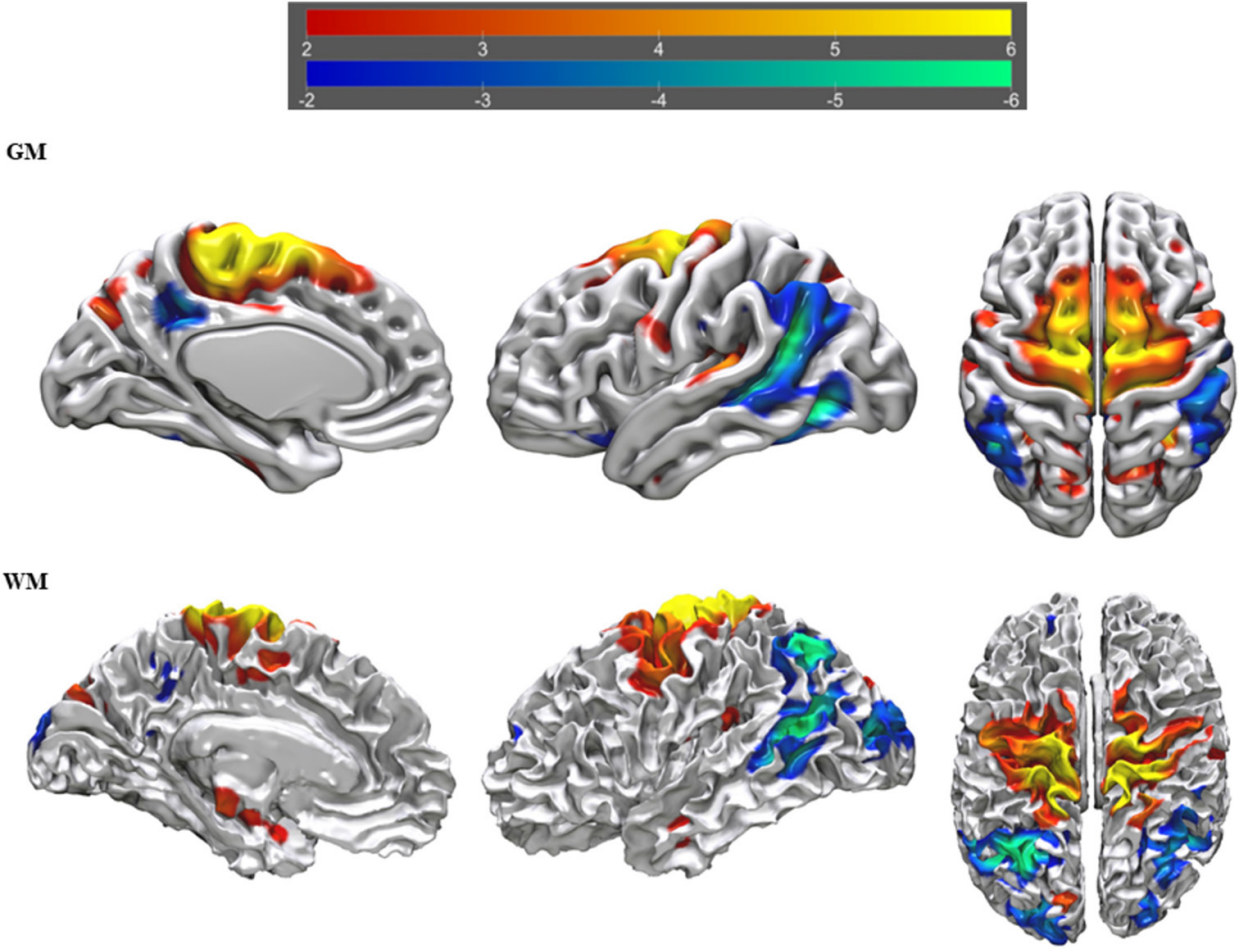
tIVA‐13 threshold was set at z‐score > 2. From left to right are displayed brain plots of tIVA‐GM 13 of the left hemisphere in medial view, of the left hemisphere in lateral view and of both hemispheres in superior view (top) and brain plots of tIVA‐WM 13 of the left hemisphere in medial view, of the left hemisphere in lateral view and of both hemispheres in superior view. Regions with increased GM and WM are represented with warm colours, whereas regions with decreased GM and WM are represented with cold colours.

From the backward stepwise regression analysis, we extracted one significant winning model for the prediction of DT scores total, which included the least number of components (*R* = 0.252, *R* ^2^ = 0.064, adjusted *R* ^2^ = 0.044, RMSE = 8.956, *F* = 3.323, *p* = 0.012). This model included four tIVA components with a significant *p* value (tIVA4 = 0.032; tIVA5 = 0.020; tIVA12 = 0.047; tIVA13 = 0.040). See Figures 2, 3, 4, 5 for a visual representation of the network areas (see Tables S1–S4 for their anatomical denominations). It is worth noting to mention that, when age is included as a covariate, the results remain the same.

**FIGURE 6:**
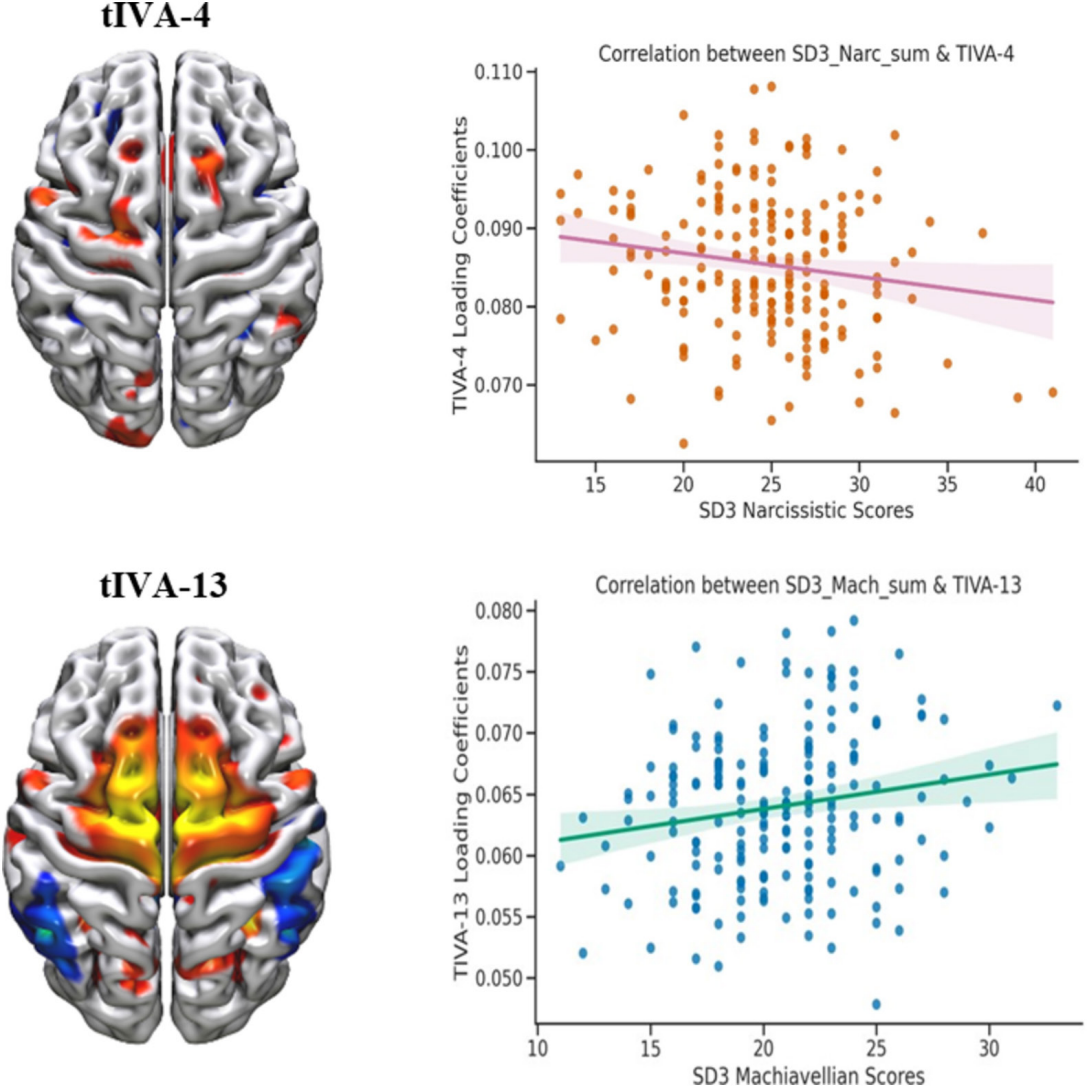
Brain circuits correlating with narcissism and Machiavellianism. Top: Brain plot depicting the tIVA‐4 component (left) and its correlation plot showing a negative association with narcissism scores (right). Bottom: Brain plot for the tIVA‐13 component (left) and its correlation plot showing a positive association with Machiavellianism scores (right).

Upon subsequent analyses, we conducted correlation analyses (see Figures S6 and S7 for the distributions of Dark Triad traits and for the choice of the statistical test based on these distributions) to examine the relationship between the 4 tIVA estimated components and the specific Dark Triad subscales (psychopathy, narcissism and Machiavellianism). Interestingly, we found that the tIVA‐4 component was negatively correlated with narcissistic traits (Spearman's *ρ* = −0.148, *p* < 0.037), whereas the tIVA‐13 component was positively correlated with Machiavellian traits (Spearman's *ρ* = 0.160, *p* < 0.024; Figure 6).

### 3.3 Relationship Between Sex, Age and Dark Triad Traits

Participants were equally distributed by sex (96 females and 105 males), and the range of age in years went from 20 to 70. Participants had an average overall DT total score of 63.90 (SD = 9.16) out of a maximum of 135 on the SD3 questionnaire, with an average score of 20.74 (SD = 3.80), 24.50 (SD = 4.72) and 18.66 (SD = 4.27) on the Machiavellianism, narcissism and psychopathy subscales, respectively. Analysing the results separately for males and females, we found that males had an average DT total score of 65.73 and females had an average DT total score of 61.89. The SD3 scores of male participants (M = 65.73, SD = 9.20) were statistically significantly higher than the SD3 scores obtained by female participants (M = 61.89, SD = 8.73). For what concerns age effect on the DT, Pearson's correlation revealed that there was no significant association between age and Machiavellianism (*r* = 0.104; *p* = 0.142), between age and narcissism (*r* = −0.062; *p* = 0.384), between age and psychopathy (*r* = −0.120; *p* = 0.090) and between age and DT total (*r* = −0.045; *p* = 0.530).

## 4 Discussion

In our study, we investigated the neural correlates of the DT traits (narcissism, Machiavellianism and psychopathy) by using the unsupervised machine learning technique tIVA (Adali, Levin‐Schwartz, and Calhoun 2015a, 2015b) to determine the joint contribution of GM and WM concentration. To our knowledge, this is the first time that the tIVA algorithm has been applied to unravel the neurobiological underpinnings of the DT.

Through the utilization of tIVA, we were able to decompose, in a data‐driven perspective, the brain into independent neural circuits, which provided a more biologically plausible representation of the structural organization of the brain compared to traditional anatomical parcellations (Ghomroudi, Scaltritti, and Grecucci 2023; Grecucci et al. 2022). Our findings revealed significant associations between specific neural circuits and the DT. Of the15 naturally independent neural networks detected by the tIVA algorithm, four were predictive of DT traits. Specifically, the tIVA‐4 component comprises areas within the reward network, including the thalamus, caudate, anterior cingulate and prefrontal regions (see Table S1). The tIVA‐12 component encompasses regions within the executive network, predominantly involving prefrontal and posterior areas (see Table S3). The tIVA‐13 component consists of regions within the DMN, such as the angular gyrus, the precuneus and the posterior cingulate cortex (see Table S4). However, unexpected findings were observed with the tIVA‐5 component, which overlaps with areas of the visual network, primarily located in the occipital and temporal lobes (see Table S2). Below, we discuss our findings and their implications.

### 4.1 The Role of Reward, Default and Executive Networks in the Dark Triad

Interestingly, in examining the individual subscales of the DT and their relationships with the predicted neural networks, we noticed specific patterns. The tIVA‐4 circuit correlated with narcissistic traits, and the tIVA‐13 circuit was specifically linked to Machiavellian traits (see Figure 6). Furthermore, the tIVA‐4 component showed a reduced GM and WM concentration, whereas the tIVA‐13 component exhibited an increase in GM and WM concentration (see Figure 6). This distinction is noteworthy.

Narcissistic traits, characterized by the absence of certain qualities such as self‐control and empathy (Jornkokgoud et al. 2023; Vazire and Funder 2006) as well as by the relentless pursuit of rewards and attention to maintain self‐esteem and grandiosity (Buss and Chiodo 1991; Chester et al. 2016; Lakey et al. 2008), are well documented. The observed reduction in brain concentration in areas related to reward and emotion regulation might reflect these deficits (Decety and Lamm 2006; Singer and Lamm 2009). In contrast, the heightened thinking strategies linked to the manipulative nature of Machiavellian individuals (Jones and Paulhus 2011) could be explained by the elevated brain concentration in specific regions involved in decision making, evaluating pros and cons and formulating strategic plans for achieving long‐term goals (Ali, Sousa Amorin, and Chamorro‐Premuzic 2009; Barlow, Qualter, and Stylianou 2010; Wilson, Near, and Miller 1996).

Specifically, in terms of narcissism, our findings suggest that the anterior insula plays a crucial role in the constellation of traits displayed by narcissistic individuals. The structural alterations observed in the insular regions align with previous research that emphasizes the involvement of the insula in empathy and emotion regulation (Decety and Lamm 2006; Singer and Lamm 2009). Specifically, we observed reduced GM concentration in the right anterior insula, which was associated with higher levels of narcissism. This finding implies diminished empathic abilities in narcissistic individuals (Fan et al. 2011). Moreover, the decreased volume of GM and WM concentration in frontal‐paralimbic brain regions provides additional support for the notion that structural alterations contribute to narcissistic traits (Schulze et al. 2013). As a matter of fact, Chester et al. (2016) found that grandiose narcissistic individuals had a frontostriatal weakened connectivity, suggesting that it might reflect their pursual of external affirmation as a way to make up for the gap between their actual and expected self‐rewards.

In relation to Machiavellianism, our findings indicate significant variations in several brain regions, including the basal ganglia, PFC, insula, right hippocampus, left parahippocampal gyrus and areas of the DMN such as the angular gyrus, the precuneus and the PCC. These results are consistent with prior research (Collins and Persinger 2014; Nestor et al. 2013; Verbeke et al. 2011) that suggests that individuals high in Machiavellianism display altered neural functioning in regions associated with social cognition and emotional processing. Furthermore, our study demonstrates an altered involvement of the cingulate gyrus, supporting the findings of Bereczkei et al. (2013), who discovered increased activation in the ACC among individuals high in Machiavellianism during a trust game. This suggests that the cingulate gyrus plays a role in the emotional conflict experienced by individuals with high Machiavellian tendencies during social interactions. The increased concentration of GM and WM in these areas might suggest a relationship with the heightened thinking strategies of Machiavellians to manipulate others and successfully achieve their objectives. These findings open avenues for future research to further investigate this hypothesis.

Regarding the investigation of psychopathy, our study did not reveal a specific neural circuit strongly associated with this trait. Two plausible explanations may account for this outcome. Firstly, it is conceivable that the range of psychopathic scores within the study sample may not have exhibited as much variability as observed for the other two DT sub‐traits, potentially contributing to the absence of statistically significant findings. Secondly, it is possible that psychopathy does not solely rely on one circuit, but rather on a combination of the circuits under investigation. This latter hypothesis finds support in an unexpected observation: the involvement of the tIVA‐5 component, which overlapped with brain regions associated with the visual network. Existing literature (De Oliveira‐Souza et al. 2008; Tiihonen et al. 2008) suggests an imbalance between posterior and anterior brain regions in favour of the former in individuals with high psychopathic traits, potentially leading to reduced activity in the latter. Future studies may consider further investigation into this hypothesis.

### 4.2 Sex Differences in the Dark Triad

Furthermore, our analyses revealed that male participants had significantly higher scores on the DT traits as opposed to female participants. The significant sex differences observed align with previous research (Grijalva et al. 2015; Cale and Lilienfeld 2002; Klimstra et al. 2020; Krampen et al. 1990; Muris et al. 2017; Nicholls et al. 2005; Vize et al. 2018). This suggests that males tend to exhibit higher levels of DT traits compared to females. Recognizing and understanding sex differences within the DT can have implications for various domains, particularly in the context of psychology and interpersonal relationships (Jones and Paulhus 2014).

### 4.3 Implications of Understanding the Neural Correlates of the Dark Triad

In recent years, a consensus has emerged in research advocating biological interventions to reduce antisocial and aggressive behaviour (Beauchaine et al. 2008; Hübner and White 2016; Raine et al. 2015). A better comprehension of the neural correlates of the DT is pivotal in informing such interventions for individuals exhibiting pathological narcissistic, Machiavellian and psychopathic traits, which are often associated with antisocial and aggressive behaviour (McDonald, Donnellan, and Navarrete 2012; Paulhus and Williams 2002). For example, studies on non‐invasive brain stimulation (NIBS), a technique involving the application of electrical or magnetic fields to specific brain regions to modulate their activity, demonstrate its potential in reducing antisocial and aggressive tendencies and enhancing empathy and moral judgement by targeting areas of the PFC (Choy 2021; Choy, Raine, and Hamilton 2018; Sergiou et al. 2020). Therefore, one of the implications of understanding the brain networks underlying the DT could be the targeted application of NIBS to address the detrimental behaviours associated with the DT. However, research is needed to explore this application thoroughly and its ethical implications.

## 5 Conclusions and Limitations

The present study offers new insights into the neurobiology of the DT traits by utilizing a data fusion approach that combines unsupervised machine learning and traditional backward stepwise regression. This methodology allowed us to dissect the brain of our participants into independent neural circuits, revealing the distributed nature of DT traits across various brain regions. The inclusion of both GM and WM components enhanced the comprehensiveness of our findings and shed light on the structural alterations associated with these personality characteristics.

However, it is important to acknowledge some limitations of our study and to point to future directions. Firstly, we solely focused on structural brain features, and future research could explore the fusion of structural and functional MRI data, such as resting state or task‐related scans. Additionally, our study relied on self‐report measures, such as the SD3 questionnaire, which introduces the potential for response biases and social desirability effects. The accuracy of our measurements may have been influenced by participants underreporting or overreporting their DT traits. To enhance the validity of the findings, future studies should consider integrating self‐report measures with other assessment methods, such as behavioural observations or clinician ratings. Moreover, despite the focus of this study was on subclinical traits and, therefore, our sample comprised individuals who did not exhibit exceptionally high levels of DT traits, in future research, it may be worthwhile to compare individuals with high DT scores to those with low DT scores, allowing for a more comprehensive examination. Furthermore, although our sample size was larger than previous studies in this domain, larger samples would be beneficial to confirm and validate our results. Lastly, combining unsupervised and supervised machine learning techniques could facilitate the development of predictive models for individual differences in DT traits using GM and WM features, allowing for a generalization to the whole population because these models could be tested on new unobserved cases (Grecucci et al. 2022).

Ultimately, our study represents an application of data fusion and machine learning in examining the DT traits. Overall, these findings deepen our understanding of the DT by highlighting sex differences, trait stability across age and the neural correlates of specific traits. The insights gained might have important implications for clinical intervention strategies and future research directions, ultimately contributing to a more comprehensive understanding of the DT and its impact on individuals and society.

## Author Contributions

**Richard Bakiaj:** conceptualization, data curation, formal analysis, investigation, methodology, project administration, software, validation, visualization, writing – original draft. **Clara Isabel Pantoja Muñoz:** software, visualization. **Andrea Bizzego:** formal analysis, methodology, visualization. **Alessandro Grecucci:** conceptualization, data curation, formal analysis, investigation, methodology, project administration, software, supervision, validation, visualization.

## Conflicts of Interest

The authors declare no conflicts of interest.

### Peer Review

The peer review history for this article is available at https://www.webofscience.com/api/gateway/wos/peer‐review/10.1111/ejn.16674.

## Supporting information


**Table S1** Descriptive statistics of the SD3.
**Table S2.** Normal ranges for the Dark Triad traits.
**Table S3.** Brain areas for tIVA‐4.
**Table S4.** Brain areas for tIVA‐5.
**Table S5.** Brain areas for tIVA‐12.
**Table S6.** Brain areas for tIVA‐13.
**Figure S6.** Histograms with kernel density estimate (KDE) plots to visualize the distribution of the data.
**Figure S7.** QQ plots for assessing normality of SD3 dimensions.
**Table S7.** Normality test for Dark Triad traits.

## Data Availability

The dataset analysed during the current study is available in the MPI‐Leipzig Mind‐Brain‐Body repository (https://openneuro.org/datasets/ds000221/versions/00002). The complete LEMON and N&C Data can be downloaded through this link (https://openneuro.org/datasets/ds000221/versions/00002/download). All data were downloaded using S3: aws s3 sync ‐‐no‐sign‐request s3://openneuro.org/ds000221 ds000221‐download/ (accessed 1 November 2022).
