# Supplementary material for: Unmasking the Dark Triad: A Data Fusion Machine Learning Approach to Characterize the Neural Bases of Narcissistic, Machiavellian and Psychopathic Traits
Source: Eur J Neurosci. 2025 Jan 22;61(2):e16674. doi: 10.1111/ejn.16674 (PMC11754945; doi:10.1111/ejn.16674)
Supplement: Supplementary file 1 — Table S1 Descriptive statistics of the SD3. Table S2. Normal ranges for the Dark Triad traits. Table S3. Brain areas for tIVA‐4. Table S4. Brain areas for tIVA‐5. Table S5. Brain areas for tIVA‐12. Table S6. Brain areas for tIVA‐13. Figure S6. Histograms with kernel density estimate (KDE) plots to visualize the distribution of the data. Figure S7. QQ plots for assessing normality of SD3 dimensions. Table S7. Normality test for Dark Triad traits. [file EJN-61-0-s001.docx]

**Supplementary Figures and Tables**

**Table 1. Descriptive Statistics of the SD3**

|  | **SD3_Mach** | **SD3_Narc** | **SD3_Psycho** | **SD3_Total** |
| --- | --- | --- | --- | --- |
| **Mean** | 20.74 | 24.5 | 18.66 | 63.9 |
| **Std. Deviation** | 3.81 | 4.72 | 4.27 | 9.16 |
| **Std. Error** | 0.27 | 0.33 | 0.3 | 0.65 |
| **Min** | 11 | 13 | 9 | 43 |
| **Max** | 33 | 41 | 31 | 95 |
| **Range** | 22 | 28 | 22 | 52 |
| **95% CI Lower** | 20.21 | 23.85 | 18.07 | 62.63 |
| **95% CI Upper** | 21.26 | 25.15 | 19.25 | 65.16 |

**Table 2. Normal Ranges for the Dark Triad traits**

| **Trait** | **Mean** | **Standard Deviation** | **Score above this is outside the normal range** |
| --- | --- | --- | --- |
| **Machiavellianism** | 3.1 | 0.76 | > 3.86 |
| **Narcissism** | 2.8 | 0.88 | > 3.68 |
| **Psychopathy** | 2.4 | 1 | > 3.40 |

**Table 3: Brain areas for tIVA-4**

| **Independent component tIVA4-GM positive values** | | | |
| --- | --- | --- | --- |
| **Area** | **Brodmann Area** | **Volume (cc)** | **MNI (x, y, z)** |
| Lentiform Nucleus | * | 3.8/4.5 | (-18, 10, 0)/(19, 10, -1) |
| Extra-Nuclear | 13 | 2.7/3.8 | (-15, 13, 1)/(16, 13, 0) |
| Thalamus | * | 3.6/4.7 | (-12, -22, 9)/(13, -19, 10) |
| Caudate | * | 2/2.9 | (-12, 13, 4)/(13, 13, 3) |
| Insula | * | 0.3/0.8 | (-34, 6, -6)/(36, -1, -3) |
| Claustrum | * | 0.1/0.3 | (-31, 6, -3)/(33, 3, -4) |
| Culmen | * | 1.5/0.0 | (-37, -36, -34)/(0, 0, 0) |
| Pyramis | * | 0.0/1.2 | (0, 0, 0)/(15, -84, -42) |
| Uvula | * | 0.0/0.4 | (0, 0, 0)/(12, -81, -42) |

| **Independent component tIVA4-GM negative values** | | | |
| --- | --- | --- | --- |
| **Area** | **Brodmann Area** | **Volume (cc)** | **MNI (x, y, z)** |
| Posterior Cingulate | 30, 31 | 2.3/2.4 | (-21, -58, 6)/(22, -58, 7) |
| Extra-Nuclear | * | 0.6/0.9 | (-21, -55, 9)/(22, -54, 6) |
| Sub-Gyral | * | 2.0/2.4 | (-12, -31, 45)/(21, -58, 12) |
| Uncus | 20, 28, 34, 36, 38 | 1.2/0.8 | (-19, 4, -39)/(19, 3, -34) |
| Cingulate Gyrus | 24, 31, 32 | 3.5/2.8 | (-12, -28, 42)/(12, -30, 43) |
| Middle Temporal Gyrus | 21, 22 | 4.7/0.7 | (-51, -40, 3)/(54, -25, -4) |
| Lateral Ventriculus | * | 0.1/0.7 | (-27, -58, 6)/(27, -52, 6) |
| Lingual Gyrus | 18 | 0.5/0.8 | (-21, -55, 3)/(22, -55, 1) |
| Parahippocampal Gyrus | 30 | 0.1/0.3 | (-18, -52, 3)/(21, -51, 3) |
| Paracentral Lobule | 5, 6, 31 | 0.8/0.8 | (-7, -24, 45)/(9, -28, 46) |
| Superior Temporal Gyrus | 13, 22, 38 | 1.0/1.1 | (-22, 7, -39)/(48, -42, 15) |
| Inferior Frontal Gyrus | 45, 46, 47 | 1.2/0.2 | (-15, 15, -25)/(16, 15, -24) |
| Rectal Gyrus | 11 | 1.0/0.8 | (-12, 12, -24)/(13, 12, -24) |
| Medial Frontal Gyrus | 6, 8, 9, 10, 25 | 2.2/3.5 | (-10, 15, -21)/(6, 45, 27) |
| Cuneus | 18, 30 | 0.5/0.5 | (-15, -64, 4)/(16, -66, 4) |
| Orbital Gyrus | 47 | 0.1/0.2 | (-15, 22, -27)/(12, 19, -27) |
| Precuneus | 31 | 0.8/0.3 | (-12, -64, 16)/(9, -36, 46) |
| Insula | 13 | 0.2/1.3 | (-40, 19, 7)/(39, -22, 19) |
| Declive | * | 1.1/0.0 | (-28, -61, -19)/(0, 0, 0) |

| **Independent component tIVA4-WM positive values** | | | |
| --- | --- | --- | --- |
| **Area** | **Brodmann Area** | **Volume (cc)** | **MNI (x, y, z)** |
| Sub-Gyral | * | 22.7/24.7 | (-27, -43, 25)/(24, -42, 27) |
| Extra-Nuclear | * | 12.7/12.5 | (-31, -45, 19)/(28, -42, 25) |
| Cingulate Gyrus | 23, 24, 31, 32 | 6.3/7.2 | (-16, -39, 27)/(19, -40, 27) |
| Insula | 13 | 0.6/0.8 | (-31, -39, 19)/(36, -42, 19) |
| Superior Temporal Gyrus | 22, 41 | 3.5/2.8 | (-36, -45, 16)/(36, -39, 16) |
| Inferior Parietal Lobule | 40 | 4.7/0.7 | (-34, -48, 25)/(37, -42, 28) |
| Posterior Cingulate | 23, 29 | 0.1/0.7 | (-4, -31, 22)/(9, -36, 22) |
| Precuneus | 31 | 0.5/0.8 | (-21, -49, 30)/(22, -49, 30) |
| Lateral Ventricle | * | 2.6/2.8 | (-19, -45, 16)/(16, -37, 21) |
| Middle Temporal Gyrus | 39 | 0.1/0.3 | (-37, -45, 4)/(39, -43, 4) |
| Transverse Temporal Gyrus | 41 | 1.0/1.1 | (-36, -39, 10)/(36, -33, 10) |
| Supramarginal Gyrus | 40 | 1.2/0.2 | (-36, -48, 30)/(40, -42, 31) |
| Anterior Cingulate | 24, 33 | 1.0/0.8 | (-3, 9, 24)/(3, 9, 24) |
| Postcentral Gyrus | 2 | 2.2/3.5 | (-37, -21, 30)/(40, -30, 31) |
| Precentral Gyrus | * | 0.5/0.5 | (-34, -15, 28)/(37, -15, 30) |
| Caudate | * | 0.8/0.3 | (-16, -7, 25)/(33, -40, 1) |
| Middle Occipital Gyrus | 18 | 0.2/1.3 | (-24, -78, 6)/(0, 0, 0) |

**Table 4: Brain areas for tIVA-5**

| **Independent component tIVA5-GM positive values** | | | |
| --- | --- | --- | --- |
| **Area** | **Brodmann Area** | **Volume (cc)** | **MNI (x, y, z)** |
| Posterior Cingulate | 23, 29, 30, 31 | 4.4/4.7 | (-19, -61, 6)/(21, -61, 7) |
| Extra-Nuclear | * | 0.6/1.8 | (-24, -58, 6)/(24, -58, 4) |
| Cuneus | 7, 17, 18, 19, 23, 30 | 14.7/15.2 | (-15, -69, 7)/(13, -72, 10) |
| Sub-Gyral | * | 0.7/2.2 | (-16, -56, 12)/(22, -60, 12) |
| Lateral Ventricle | * | 0.3/1.0 | (-27, -61, 6)/(27, -61, 6) |
| Precuneus | 7, 19, 23, 31 | 2.2/2.8 | (0, -73, 21)/(3, -76, 21) |
| Lingual Gyrus | 17, 18, 19 | 7.6/8.8 | (-16, -55, 3)/(21, -57, 1) |
| Parahippocampal Gyrus | 18, 30 | 0.5/0.9 | (-21, -54, 3)/(21, -51, 1) |
| Inferior Occipital Gyrus | 17, 18, 19 | 0.7/1.5 | (-13, -93, -13)/(15, -91, -13) |
| Middle Occipital Gyrus | 18, 19 | 3.6/4.7 | (-36, -81, 10)/(22, -88, -13) |
| Middle Temporal Gyrus | 39 | 0.3/0.4 | (-36, -81, 16)/(40, -76, 10) |
| Superior Temporal Gyrus | 22, 39 | 0.0/0.3 | (0, 0, 0)/(54, -57, 10) |

| **Independent component tIVA5-WM positive values** | | | |
| --- | --- | --- | --- |
| **Area** | **Brodmann Area** | **Volume (cc)** | **MNI (x, y, z)** |
| Cuneus | 7, 17, 18, 19, 23, 30 | 11.6/13.0 | (-15, -78, 16)/(15, -78, 19) |
| Precuneus | 7, 18, 19, 31 | 1.8/3.1 | (-15, -75, 19)/(12, -75, 19) |
| Posterior Cingulate | 30, 31 | 2.5/3.1 | (-21, -67, 13)/(22, -67, 15) |
| Middle Occipital Gyrus | 18, 19 | 3.1/4.4 | (-21, -87, 10)/(21, -85, 13) |
| Sub-Gyral | * | 4.5/8.2 | (-24, -69, 16)/(19, -73, 21) |
| Lingual Gyrus | 17, 18, 19 | 9.0/7.2 | (-16, -82, 1)/(19, -82, 1) |
| Extra-Nuclear | * | 1.2/2.2 | (-27, -64, 10)/(28, -67, 9) |
| Lateral Ventricle | * | 0.4/0.9 | (-27, -69, 3)/(28, -61, 9) |
| Parahippocampal Gyrus | 18, 30 | 0.1/0.3 | (-22, -54, 3)/(25, -57, 1) |
| Middle Temporal Gyrus | 22 | 0.3/0.8 | (-33, -67, 12)/(30, -78, 16) |
| Inferior Occipital Gyrus | 18, 19 | 0.4/0.4 | (-25, -88, -13)/(31, -82, -10) |
| Superior Temporal Gyrus | 39 | 0.0/0.3 | (0, 0, 0)/(37, -55, 12) |

| **Independent component tIVA5-WM negative values** | | | |
| --- | --- | --- | --- |
| **Area** | **Brodmann Area** | **Volume (cc)** | **MNI (x, y, z)** |
| Sub-Gyral | * | 0.1/1.2 | (-30, -63, 36)/(33, -60, 39) |
| Inferior Parietal Lobule | 7, 39 | 0.1/0.6 | (-34, -64, 37)/(36, -63, 39) |
| Angular Gyrus | 39 | 0.0/0.2 | (0, 0, 0)/(36, -60, 36) |
| Precuneus | 7, 19, 39 | 0.6/0.3 | (-31, -67, 37)/(33, -66, 39) |
| Superior Parietal Lobule | 7 | 0.2/0.3 | (-31, -67, 43)/(30, -57, 45) |

**Table 5: Brain areas for tIVA-12**

| **Independent component tIVA12-GM positive values** | | | |
| --- | --- | --- | --- |
| **Area** | **Brodmann Area** | **Volume (cc)** | **MNI (x, y, z)** |
| Middle Frontal Gyrus | 9, 11, 47 | 5.4/5.5 | (-28, 43, -13)/(30, 42, -15) |
| Superior Frontal Gyrus | 11 | 1.5/2.8 | (-25, 46, -15)/(27, 45, -15) |
| Sub-Gyral | * | 4.5/3.2 | (-31, -57, 42)/(40, -33, 45) |
| Inferior Parietal Lobule | 7, 39, 40 | 3.6/2.6 | (-34, -55, 45)/(37, -36, 43) |
| Inferior Frontal Gyrus | 9, 11, 44, 47 | 2.9/2.8 | (-46, 9, 24)/(28, 31, -16) |
| Anterior Cingulate | 24, 32, 33 | 0.5/2.8 | (0, 24, 22)/(3, 30, 19) |
| Precuneus | 7, 19, 39 | 1.7/2.4 | (-30, -64, 39)/(28, -66, 36) |
| Superior Parietal Lobule | 5, 7 | 0.9/0.2 | (-28, -55, 45)/(21, -45, 64) |
| Declive | * | 0.0/2.2 | (0, 0, 0)/(13, -79, -30) |
| Postcentral Gyrus | 2, 3, 5, 7, 40 | 0.3/2.8 | (-22, -43, 61)/(37, -31, 42) |
| Extra-Nuclear | * | 0.1/0.3 | (-3, 24, 19)/(3, 24, 19) |
| Uvula | * | 0.0/0.8 | (0, 0, 0)/(15, -79, -34) |
| Middle Occipital Gyrus | 19 | 1.5/0.0 | (-30, -87, 7)/(0, 0, 0) |
| Cingulate Gyrus | 24, 32 | 0.4/0.6 | (-1, 13, 28)/(3, 22, 30) |
| Pyramis | * | 0.0/0.6 | (0, 0, 0)/(9, -79, -33) |
| Superior Temporal Gyrus | 22, 38 | 0.0/2.2 | (0, 0, 0)/(40, 16, -33) |
| Precentral Gyrus | 3, 9, 44 | 0.5/0.6 | (-49, 9, 15)/(39, 18, 39) |
| Cuneus | 18, 19 | 0.6/0.2 | (-19, -88, 24)/(18, -85, 33) |
| Middle Temporal Gyrus | 21, 38 | 0.1/0.7 | (-34, -79, 15)/(40, 7, -39) |
| Supramarginal Gyrus | 40 | 0.1/0.3 | (-37, -51, 37)/(55, -43, 34) |

| **Independent component tIVA12-GM negative values** | | | |
| --- | --- | --- | --- |
| **Area** | **Brodmann Area** | **Volume (cc)** | **MNI (x, y, z)** |
| Lingual Gyrus | 17, 18 | 0.1/4.2 | (-1, -88, -16)/(7, -90, -13) |
| Middle Occipital Gyrus | 18, 19, 37 | 0.0/2.8 | (0, 0, 0)/(40, -75, -15) |
| Posterior Cingulate | 23, 29, 30, 31 | 2.9/1.3 | (0, -51, 22)/(3, -52, 19) |
| Inferior Occipital Gyrus | 17, 18, 19 | 0.0/3.3 | (0, 0, 0)/(42, -75, -9) |
| Fusiform Gyrus | 19, 37 | 0.0/1.9 | (0, 0, 0)/(39, -72, -18) |
| Cingulate Gyrus | 31 | 0.6/0.7 | (-3, -51, 22)/(1, -52, 27) |
| Inferior Temporal Gyrus | 37 | 0.0/0.6 | (0, 0, 0)/(43, -73, -4) |
| Sub-Gyral | * | 0.0/1.1 | (0, 0, 0)/(40, -69, -15) |
| Precuneus | 7, 31 | 0.7/1.0 | (-1, -52, 30)/(4, -52, 30) |
| Declive | * | 0.0/1.0 | (0, 0, 0)/(39, -73, -22) |
| Extra-Nuclear | * | 0.3/0.3 | (-3, -43, 9)/(3, -43, 9) |
| Cerebellar Tonsil | * | 0.3/0.1 | (0, -58, -43)/(21, -51, -52) |
| Cuneus | * | 0.0/0.3 | (0, 0, 0)/(10, -99, -7) |
| Culmen | * | 0.0/0.5 | (0, 0, 0)/(27, -46, -22) |
| Uncus | 28, 36 | 0.3/0.0 | (-19, -1, -34)/(0, 0, 0) |

| **Independent component tIVA12-WM positive values** | | | |
| --- | --- | --- | --- |
| **Area** | **Brodmann Area** | **Volume (cc)** | **MNI (x, y, z)** |
| Anterior Cingulate | 24, 32 | 0.3/2.2 | (-3, 30, 13)/(9, 30, 12) |
| Pyramis | * | 0.6/1.6 | (-21, -63, -39)/(16.5, -6.750000e+01, -36) |
| Extra-Nuclear | * | 2.6/2.6 | (-1, 25, 9)/(9, 27, 15) |
| Sub-Gyral | * | 0.8/0.8 | (-24, -48, 51)/(12, 33, 10) |
| Declive | * | 0.0/1.9 | (0, 0, 0)/(12, -66, -30) |
| Cuneus | 7, 18, 19 | 1.0/1.2 | (-10, -82, 30)/(13, -79, 33) |
| Precuneus | 7, 19, 31 | 0.4/1.8 | (-24, -51, 54)/(19, -78, 34) |
| Lateral Ventricle | * | 0.3/0.4 | (-4, 22, 9)/(9, 24, 10) |
| Cerebellar Tonsil | * | 1.3/0.4 | (-24, -60, -40)/(19, -63, -42) |
| Inferior Semi-Lunar Lobule | * | 0.1/0.6 | (-18, -66, -45)/(19, -66, -45) |
| Culmen | * | 0.4/0.3 | (-22, -5, -34)/(12, -61, -27) |
| Middle Occipital Gyrus | 19 | 0.4/0.0 | (-25, -85, 15)/(0, 0, 0) |

| **Independent component tIVA12-WM negative values** | | | |
| --- | --- | --- | --- |
| **Area** | **Brodmann Area** | **Volume (cc)** | **MNI (x, y, z)** |
| Middle Occipital Gyrus | 18, 19, 37 | 0.4/9.2 | (-27, -69, 1)/(24, -84, -1) |
| Sub-Gyral | * | 0.7/6.0 | (-27, 19, 39)/(27, -84, -4) |
| Lingual Gyrus | 17, 18, 19 | 0.9/8.3 | (-24, -88, -12)/(21, -87, -1) |
| Cuneus | 17, 18, 30 | 0.1/7.0 | (-15, -66, 4)/(21, -90, 1) |
| Inferior Occipital Gyrus | 17, 18, 19 | 0.4/2.4 | (-28, -88, -13)/(31, -81, -10) |
| Inferior Parietal Lobule | 7, 39, 40 | 0.1/1.0 | (-46, -25, 27)/(34, -63, 39) |
| Angular Gyrus | 39 | 0.0/0.4 | (0, 0, 0)/(34, -60, 36) |
| Posterior Cingulate | 23, 30, 31 | 0.4/0.5 | (-7, -58, 22)/(28, -72, 7) |
| Inferior Temporal Gyrus | * | 0.0/0.3 | (0, 0, 0)/(40, -72, -4) |
| Precuneus | 19, 31, 39 | 0.6/1.6 | (-10, -58, 25)/(34, -66, 36) |
| Inferior Frontal Gyrus | * | 0.0/1.2 | (0, 0, 0)/(45, 31, 4) |
| Cingulate Gyrus | 31, 32 | 0.4/0.6 | (-10, -55, 28)/(10, -55, 28) |
| Medial Frontal Gyrus | 6, 9 | 0.1/1.9 | (-13, 55, -3)/(15, 16, 52) |
| Middle Frontal Gyrus | 8 | 0.4/0.7 | (-28, 22, 42)/(31, 19, 43) |
| Superior Parietal Lobule | 7 | 0.0/0.4 | (0, 0, 0)/(31, -60, 45) |
| Extra-Nuclear | * | 0.4/1.7 | (-13, -7, -1)/(31, -66, 4) |
| Middle Temporal Gyrus | * | 0.1/0.2 | (-54, -52, 4)/(40, -78, 7) |
| Lentiform Nucleus | * | 0.1/0.6 | (-13, -4, 1)/(15, -4, 3) |
| Anterior Cingulate | 32 | 0.0/0.1 | (0, 0, 0)/(9, 37, 28) |
| Precentral Gyrus | * | 0.1/0.0 | (-31, 19, 40)/(0, 0, 0) |

**Table 6: Brain areas for tIVA-13**

| **Independent component tIVA13-GM positive values** | | | |
| --- | --- | --- | --- |
| **Area** | **Brodmann Area** | **Volume (cc)** | **MNI (x, y, z)** |
| Medial Frontal Gyrus | 6 | 5.7/5.3 | (-10, -15, 70)/(3, -22, 67) |
| Middle Frontal Gyrus | 6 | 3.5/3.0 | (-16, -12, 66)/(18, -3, 66) |
| Superior Frontal Gyrus | 6, 8 | 6.0/4.5 | (-9, -10, 69)/(9, -6, 69) |
| Paracentral Lobule | 4, 5, 6, 31 | 3.3/2.5 | (-1, -33, 66)/(4, -33, 66) |
| Precentral Gyrus | 4, 6 | 4.4/5.9 | (-12, -19, 70)/(25, -24, 60) |
| Sub-Gyral | 6 | 3.6/3.7 | (-22, -10, 61)/(25, -9, 58) |
| Inferior Parietal Lobule | 40 | 0.2/0.4 | (-34, -52, 39)/(34, -55, 43) |
| Postcentral Gyrus | 3 | 0.4/0.3 | (-10, -36, 69)/(21, -28, 66) |
| Insula | 13, 41 | 0.6/0.0 | (-40, -28, 16)/(0, 0, 0) |

| **Independent component tIVA13-GM negative values** | | | |
| --- | --- | --- | --- |
| **Area** | **Brodmann Area** | **Volume (cc)** | **MNI (x, y, z)** |
| Supramarginal Gyrus | 40 | 2.4/1.9 | (-42, -57, 25)/(46, -49, 25) |
| Superior Temporal Gyrus | 13, 22, 39 | 4.0/2.5 | (-43, -54, 22)/(48, -36, 4) |
| Sub-Gyral | * | 2.4/0.4 | (-43, -58, -9)/(43, -46, 24) |
| Angular Gyrus | 39 | 0.6/0.2 | (-42, -63, 31)/(42, -57, 30) |
| Middle Temporal Gyrus | 21, 22, 39 | 2.8/0.5 | (-42, -61, 22)/(51, -36, 1) |
| Inferior Parietal Lobule | 40 | 0.9/0.8 | (-46, -49, 22)/(48, -46, 22) |
| Middle Occipital Gyrus | 19 | 0.5/0.0 | (-45, -67, -7)/(0, 0, 0) |
| Inferior Temporal Gyrus | 20, 37 | 0.6/0.0 | (-46, -60, -6)/(0, 0, 0) |
| Cingulate Gyrus | 31 | 1.5/0.2 | (-6, -46, 37)/(3, -43, 37) |
| Fusiform Gyrus | 37 | 0.5/0.0 | (-45, -54, -18)/(0, 0, 0) |
| Postcentral Gyrus | 2, 3, 4, 40 | 0.0/0.9 | (0, 0, 0)/(58, -16, 33) |
| Precuneus | 7, 31 | 0.7/0.0 | (-9, -49, 37)/(0, 0, 0) |
| Precentral Gyrus | 4 | 0.0/0.5 | (0, 0, 0)/(60, -19, 36) |

| **Independent component tIVA13-WM positive values** | | | |
| --- | --- | --- | --- |
| **Area** | **Brodmann Area** | **Volume (cc)** | **MNI (x, y, z)** |
| Medial Frontal Gyrus | 6, 32 | 7.7/7.0 | (-13, -21, 60)/(15, -21, 58) |
| Sub-Gyral | 3, 6, 8, 24 | 10.2/8.2 | (-16, -18, 60)/(18, -18, 58) |
| Middle Frontal Gyrus | 6 | 2.9/2.0 | (-16, -15, 63)/(24, -18, 58) |
| Paracentral Lobule | 3, 4, 5, 6 | 2.3/2.0 | (-12, -33, 60)/(13, -27, 52) |
| Precentral Gyrus | 4, 6 | 6.5/4.6 | (-10, -24, 67)/(25, -18, 54) |
| Superior Frontal Gyrus | 6 | 2.7/1.4 | (-10, -18, 67)/(15, -18, 69) |
| Cingulate Gyrus | 24, 31, 32 | 3.2/2.4 | (-13, -12, 51)/(15, -4, 51) |
| Postcentral Gyrus | 3, 4 | 0.9/1.0 | (-12, -39, 63)/(22, -28, 55) |
| Inferior Frontal Gyrus | 6, 9 | 0.4/0.1 | (-43, -3, 25)/(49, 1, 30) |

| **Independent component tIVA13-WM negative values** | | | |
| --- | --- | --- | --- |
| **Area** | **Brodmann Area** | **Volume (cc)** | **MNI (x, y, z)** |
| Supramarginal Gyrus | 40 | 1.2/0.3 | (-34, -55, 36)/(36, -54, 36) |
| Sub-Gyral | 39 | 9.9/3.8 | (-31, -55, 33)/(40, -51, 9) |
| Inferior Parietal Lobule | 39, 40 | 2.6/1.1 | (-37, -55, 39)/(39, -54, 40) |
| Middle Temporal Gyrus | 19, 22, 39 | 4.1/0.8 | (-42, -54, 4)/(43, -49, 6) |
| Angular Gyrus | 39 | 0.6/0.1 | (-31, -58, 36)/(39, -57, 36) |
| Superior Temporal Gyrus | 22, 39 | 1.1/0.7 | (-42, -54, 10)/(46, -49, 9) |
| Extra-Nuclear | * | 0.6/0.1 | (-33, -52, 7)/(25, -51, 18) |
| Precuneus | 19, 31 | 1.5/0.0 | (-22, -57, 33)/(0, 0, 0) |
| Middle Occipital Gyrus | 18 | 1.3/0.2 | (-24, -81, 3)/(25, -81, 3) |
| Cuneus | 17 | 0.7/0.6 | (-21, -82, 6)/(22, -78, 3) |
| Lateral Ventricle | * | 0.5/0.1 | (-31, -55, 4)/(30, -55, 10) |
| Lingual Gyrus | * | 0.1/0.3 | (-21, -82, 0)/(22, -81, 0) |

*Each tIVA component is ordered according to the model results. Inside each component, the brain areas are ordered according to the magnitude of their effect in the overall network. Note: for tIVA-4, negative values for the WM were not reported. Only areas with a volume of 0.2> are reported in the tables.*

**Figure 6. Histograms with Kernel Density Estimate (KDE) plots to visualize the distribution of the data**

**
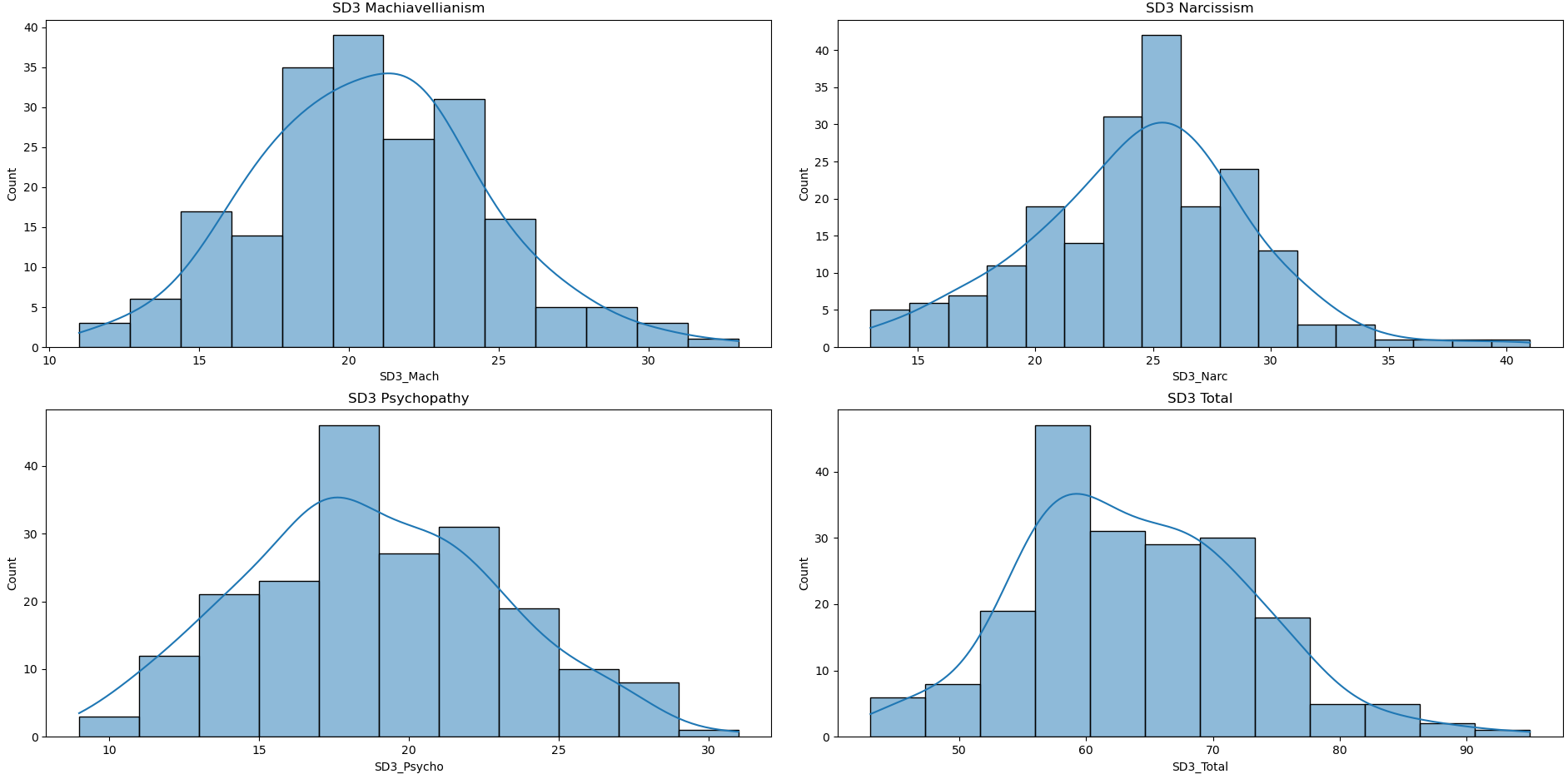
**

**Figure 7. QQ Plots for Assessing Normality of SD3 Dimensions**

**
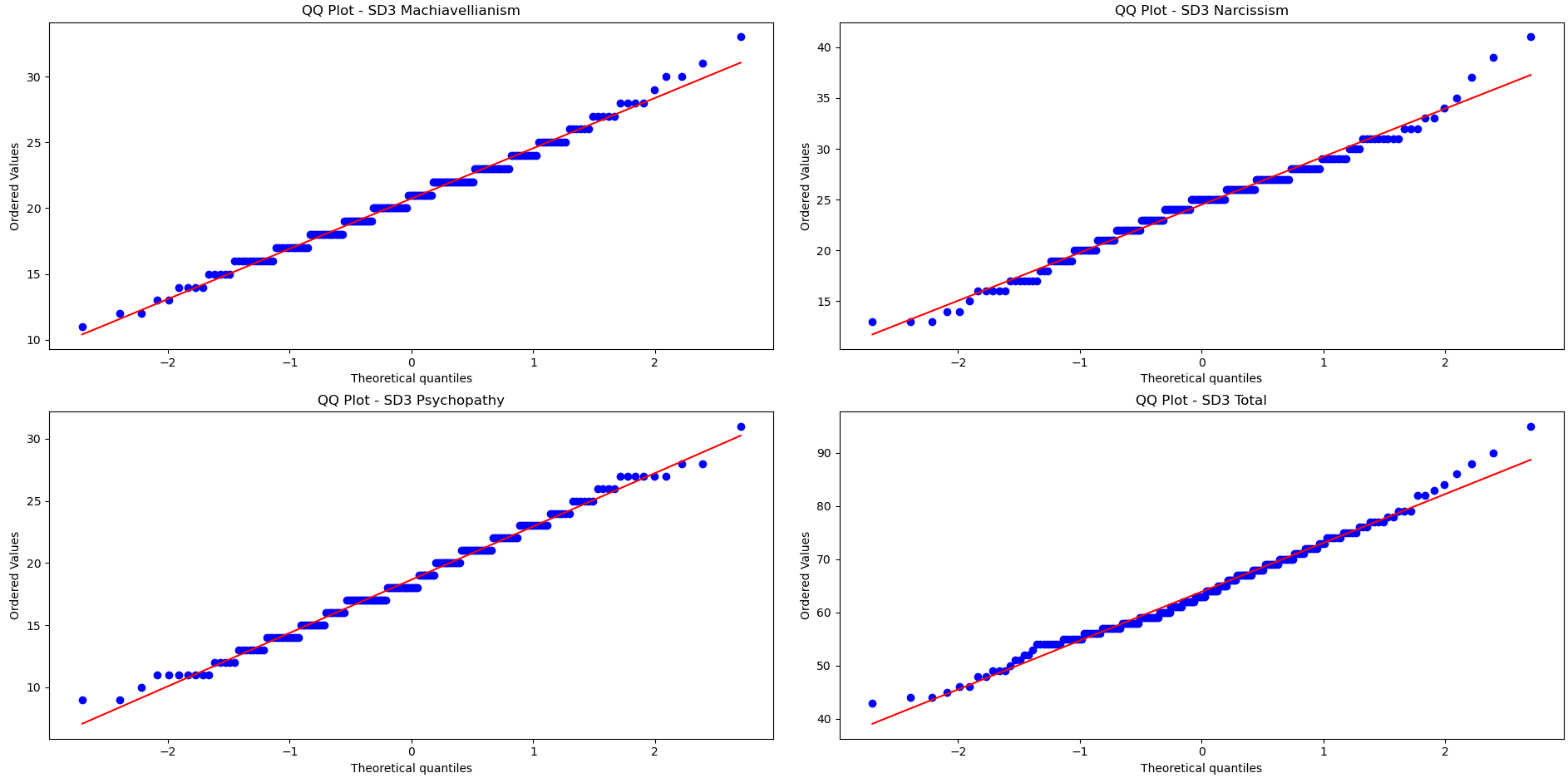
**

We have run distribution tests for both collective and individual components of the Dark Triad by using the Shapiro-Wilk test on JASP (see below Table 7).

**Table 7. Normality Test for Dark Triad Traits**

|  | **SD3_Mach** | **SD3_Narc** | **SD3_Psycho** | **SD3_Total** |
| --- | --- | --- | --- | --- |
| **Shapiro-Wilk** | 0.99 | 0.98 | 0.99 | 0.99 |
| **P-value of Shapiro-Wilk** | 0.18 | 0.02 | 0.14 | 0.069 |

The distribution of the Dark Triad total is normal. The Machiavellianism (SD3_Mach) and psychopathy (SD3_Psycho) dimensions did not significantly deviate from normality (p = 0.18, p = 0.14 respectively). However, the narcissism (SD3_Narc) dimension showed a significant deviation from normality (p = 0.02). For the SD3_Total score, the distribution did not significantly deviate from normality (p = 0.069). To address the non-normality of the dimension of narcissism, we utilized the non-parametric test Spearman’s rank correlation to explore the relationships between Machiavellianism, narcissism, and psychopathy and the 4 neural networks detected by the tIVA algorithm (tIVA-4, tIVA-5, tIVA-12, tIVA-13). We found that the tIVA-4 component was negatively correlated with narcissistic traits (r = -0.148, p < 0.037), while the tIVA-13 component was positively correlated with Machiavellian traits (r = 0.160, p < 0.024).
